# Pseudotumour Cerebri Presentation in a Child Under the Gonadotropin-Releasing Hormone Agonist Treatment

**DOI:** 10.4274/jcrpe.2212

**Published:** 2016-09-01

**Authors:** Ülkü Gül, Ayşe Kaçar Bayram, Mustafa Kendirci, Nihal Hatipoğlu, Deniz Okdemir, Hakan Gümüş, Selim Kurtoğlu

**Affiliations:** 1 Erciyes University Faculty of Medicine, Department of Pediatric Endocrinology, Kayseri, Turkey; 2 Erciyes University Faculty of Medicine, Department of Pediatric Neurology, Kayseri, Turkey

**Keywords:** Gonadotropin-releasing hormone agonist treatment, side effect, pseudotumor cerebri

## Abstract

Gonadotropin-releasing hormone analogues are common treatment option in central precocious puberty in childhood as well as in endometriosis, infertility, and prostate cancer in adults. Pseudotumor cerebri is a rare side effect observed in adults. We present the case of a girl with precocious puberty treated with triptorelin acetate who developed pseudotumor cerebri after the 4^th^ dose. She had headaches, and her blood pressure was detected to be above the 99 percentile. There were no causes underlying of hypertension such as cardiac, renal, or endocrine. Neurological examination was normal except bilateral papilledema. Cranial magnetic resonance imaging was normal. Cerebrospinal fluid (CSF) opening pressure was elevated. Triptorelin therapy was ceased and acetazolamide was applied; CSF pressure returned to normal. We observed pseudotumor cerebri after precocious puberty treatment, a finding for the first time ever seen in childhood.

**WHAT IS ALREADY KNOWN ON THIS TOPIC?**

Gonadotropin-releasing hormone (GnRH) analogues are common treatment option in central precocious puberty. Sterile abscess at the injection site, menopause-like symptoms, headache, emotional disorders, syncope, osteoporosis, vasodilatation, and peripheral edema are rare side effects in children.

**WHAT THIS STUDY ADDS?**

We presented a pediatric patient who developed pseudotumor cerebri after the treatment of precocious puberty, the side effect for the first time ever seen in childhood. So, we suggest that complaints like headache, nausea, vomiting, and double vision in pediatric patients treated with GnRH analogue should consider the presence of pseudotumor cerebri.

## INTRODUCTION

Gonadotropin-releasing hormone (GnRH) analogues are common treatment option in central precocious puberty. Synthetic leuprolide acetate and triptorelin acetate are also used safely in the treatment of endometriosis, infertility, and prostate cancer. Sterile abscess at the injection site, menopause-like symptoms, headache, emotional disorders, syncope, osteoporosis, vasodilatation, and peripheral edema are rare side effects in children (1,2). Bone pain, micturition problems, hypersensitivity (itching, skin rash, fever), gynecomastia, depression, easy and quick to anger, headache, nausea, muscle pain, joint pain, excessive sweating, fatigue, sleep disturbances, pain at the injection site, predisposition to hypertension, and thrombosis are principally observed in adults (3,4,5,6,7).

Here, we present the case of a girl with precocious puberty treated with triptorelin acetate who developed pseudotumor cerebri, a side effect for the first time ever seen in childhood.

## CASE REPORT

Nine-year-old girl was admitted because of breast development which had started 10 months before. Rapid height growth, adult body odor, and vaginal discharge are available. There was no history of drug use or chronic illness. On physical examination, weight was 34 kg [80 p, 0.8 standard deviation score (SDS)], height 138.3 cm (85 p, 1.06 SDS), arterial blood pressure 100/60 mmHg, thelarche bilateral Tanner stage 3, pubic hair stage 2, and axillary hair was not detected. Her body mass index was normal (17.7 kg/m^2^, 0.5 SDS), and she did not have history of any recent onset of weight gain. Patient’s bone age was 10.5 years. On pelvic ultrasonography (US), the length of the uterus (45 mm) and ovarian volumes (2.5 mL and 3.1 mL) were observed in pubertal size. Hypophysis magnetic resonance imaging (MRI) was normal. Gonadotropin levels were within pubertal range [follicle-stimulating hormone (FSH): 1.97 mIU/mL, luteinizing hormone: 1 mIU/mL]. Her bone age demonstrated fast progress of 1.5 years in 6 months, and annual follow-up height growth was 7.5 cm. Depot 3.75 mg of triptorelin acetate per month was started because of the early puberty. She had headaches after the 4^th^ dose, and her blood pressure was 130/80 mmHg (>99 percentile). The patient’s previous medical records revealed no history of hypertension, and her blood pressure increased after treatment. Multiple measurements showed systolic/diastolic blood pressure in the range of 130-155/85-110 mmHg. Other system examinations were normal. Complete blood count, renal function tests, and serum electrolyte levels were in normal limits. Echocardiography analysis and renal Doppler US were normal. Plasma renin activity and aldosterone levels were observed in the normal range. There was no abnormality related to patient’s neurological examination except bilateral papilledema. On cranial MRI, space-occupying mass was not observed and ventricular system was intact. The orbital section of MRI revealed bilateral optic nerve enlargement. Lumbar puncture was performed, and an elevated initial cerebrospinal fluid (CSF) opening pressure was detected (46 cm H_2_O, normal range: 15-25 H_2_O). Based on these findings, the patient was diagnosed with pseudotumor cerebri, and triptorelin therapy was ceased. Except GnRH analogue treatment, there were no any risk factors that might lead to pseudotumor cerebri such as obesity, renal failure, drugs, etc. The patient improved with treatment of acetazolamide, and the CSF pressure and fundoscopic examinations returned to normal. So, we think that the high blood pressure might be due to the increased intracranial pressure.

## DISCUSSION

We presented a patient who developed pseudotumor cerebri after treatment with a GnRH analogue, triptorelin acetate. GnRH analogues are in the form of injectable depot, administered 3.75 mg monthly or 11.25 mg every three months.

The common side effects of GnRH analogues in both adults and children are as follows: sweating, flushing, sleep disorders, psychiatric disorders such as depression, bone mineral density reduction with long-term use, and osteoporosis because of their menopause-like effects (1,3,4). Also, side effects such as headaches, muscle pain, allergy, skin eruption, sterile abscess, hypertension, hypercoagulability may occur (4,5,6). Cardiovascular effects reported in adults are associated with hypoestrogenism and hipoandrogenism (7,8). In reports, two patients with central precocious puberty who developed hypertension because of triptorelin acetate treatment are explained by the same mechanism (8,9).

Our patient was diagnosed with central precocious puberty and treated with triptorelin acetate. Under the treatment, she developed headaches and hypertension. There were no causes underlying of hypertension such as cardiac, renal, or endocrine. Neurological examination was normal except bilateral papilledema. Cranial MRI was normal. On lumbar puncture, initial CSF opening pressure was elevated. The patient was diagnosed with pseudotumor cerebri based on those findings, and triptorelin therapy was ceased. We could not find a reason to explain intracranial hypertension such as Cushing disease, hypoparathyroidism, iron deficiency anemia, obesity, or any history of drug use. The patient improved with treatment of acetazolamide, and the CSF pressure and fundoscopic examination returned to normal.

Pseudotumor cerebri is characterized by a normal neurological examination except for sixth cranial nerve paralysis and papilledema without pathological findings on brain MRI (10). While there is no reason in the majority of adult patients, an underlying cause is determined in 53-77% of pediatric cases. The annual incidence is 1/100.000 (11). Endocrine abnormalities, metabolic problems, infections, trauma, medications, and venous sinus thrombosis constitute the etiology. Among the most common causes of pseudotumor cerebri due to drugs, oral contraceptives, cyclosporine, isotretinoin, phenytoin, and steroids may be considered. The symptoms in children were headache, nausea, vomiting, blurred vision, diplopia, neck stiffness, photophobia, and retro-orbital pain. Prognosis is better in children than adults. Lumbar puncture can be reduced to the CSF pressure within normal limits and provides therapeutic effects on symptoms in most children (12). Lowering of the intracranial pressure as soon as possible is crucial in order to prevent compression of the optic nerves and vision loss. Therapeutic options of pseudotumor cerebri include medical and surgical modalities, but causal factors such as drugs and obesity should be eliminated primarily. Medical management in childhood is essential. The first-line treatment is acetazolamide. Pseudotumor cerebri has a good prognosis with medical therapy in the pediatric population (13).

In the literature, it was reported that only four adult patients developed pseudotumor cerebri after the use of GnRH analogue (14,15,16,17). One of the patients used triptorelin acetate and the others used leuprolide acetate. All patients had different disorders; however, before the GnRH analogue treatment, none of them had neither predispositions such as obesity, Addison disease, drugs etc. nor clinical findings consistent with pseudotumor cerebri. After stopping the GnRH analogue treatment and starting the convenient treatment, all patients returned to normal. The authors did not find any other reasons, thus, those clinical events were accepted as a side effect of the GNRH analogue treatment (14,15,16,17).

Pseudotumor cerebri development mechanism after GnRH treatment is not fully understood. Possible mechanism is: GnRH analogue causes a short-term increase of sex steroids after injection and a mild venous sinus thrombosis develops due to high serum levels of steroids. Also they may cause non-obstructive thrombosis of the dural venous sinuses by creating a venous hypertensive state and prevent the CSF drainage. Therefore, benign intracranial hypertension (BIH) arises (14). As well as in our case, after cessation of treatment, normalization of CSF pressure and eye examination suggests strongly the role of GnRH analogues in the BIH etiology. Complaints such as headache, nausea, vomiting, and double vision in pediatric patients treated with GnRH analogue should consider the presence of pseudotumor cerebri and fundus examination should be performed.

We presented a pediatric patient who developed pseudotumor cerebri after treatment of precocious puberty. To our knowledge, this side effect of triptorelin acetate is for the first time ever seen in childhood.

## Ethics

Informed Consent: It was taken.

Peer-review: Externally peer-reviewed.
